# Radial extracorporeal shock wave therapy improves upper limb function in chronic stroke patients: a randomized sham-controlled trial

**DOI:** 10.1016/j.clinsp.2026.101034

**Published:** 2026-07-09

**Authors:** Marta Imamura, Andre Tadeu Sugawara, Artur Cesar Aquino Santos, Jose Oliveira Siqueira, Paulo Sergio Panse Silveira, Christoph Schmitz, Linamara Rizzo Battistella

**Affiliations:** a Departamento de Medicina Legal, Bioetica, Medicina do Trabalho e Medicina Fisica e Reabilitacao, Faculdade de Medicina HCFMUSP, Universidade de São Paulo, São Paulo, SP, Brazil; b Instituto de Medicina Fisica e Reabilitacao, Hospital das Clinicas HCFMUSP, Faculdade de Medicina, Universidade de São Paulo, São Paulo, SP, Brazil; c Departmento de Patologia, Faculdade de Medicina, Universidade de São Paulo, São Paulo, SP, Brazil; d Extracorporeal Shock Wave Research Unit, Chair of Neuroanatomy, Institute of Anatomy, Faculty of Medicine, LMU Munich, Munich, Germany

**Keywords:** Upper limb, Motor recovery, Stroke, Post-stroke spasticity, Radial extracorporeal shockwave therapy, Fugl-Meyer Assessment, Randomized controlled trial

## Abstract

**Context:**

Stroke causes upper limb disability. The literature shows the effectiveness of radial Extracorporeal Shock Wave Therapy (rESWT) in reducing spasticity and promoting functional recovery of the upper limb of patients with stroke.

**Objective:**

Demonstrate the effect of rESWT on functional recovery of the upper extremity after ischemic stroke in the middle cerebral artery territory.

**Methods:**

Randomized, doubleblind, sham-controlled trial. Interventions: Weekly sessions of 5,000 rESWs (3.5 to 4.0 bar, 15 Hz), diffusely over the elbow and wrist flexors in the forearm and hand interosseous muscles for four weeks, delivered with a 36 mm device. A control treatment was conducted by a sham handpiece that did not emit the rESWs.

**Outcomes:**

Changes in Fugl-Meyer Assessment for upper limb function (FMA-UE) at four weeks after the end of the treatment was the primary outcome. Spasticity, strength, and cortical integrity by transcranial magnetic stimulation were also assessed before and four weeks after the end of the treatment .

**Results:**

23 patients were randomized, and 16 were included in the per-protocol analysis. Baseline measures were were balanced. FMA-UE was significantly different between the groups for proximal muscles (*p* = 0.004) and overall score (*p* = 0.038). Muscle strength by the Medical Research Council (MRC) score was also significantly different between both groups (*p* = 0.012). Three patients of the rESWT group and four patients of the sham rESWT group were excluded due to the use of Botulinum toxin, declined participation soon after they were randomized, or did not return four weeks after the end of treatment to follow-up. A limitation of this study is its small sample size, which limits the statistical power and the generalizability of our findings.

**Conclusion:**

Four weekly sessions of rESWT improved the functional recovery of patients with chronic ischemic stroke and absent MEPs at baseline. This suggests rESWT may act via peripheral mechanisms to improve proximal muscle function, reflected in the overall improvement of the global measure of FMA-UE.

## Highlights

• First demonstration that rESWT improves proximal upper limb function in chronic stroke patients with absent Motor Evoked Potentials (MEPs) ‒ a subgroup with traditionally poor prognosis.
• rESWT significantly enhances muscle strength (MRC scale) and functional recovery (FMA-UE) through likely peripheral mechanisms, independent of corticospinal tract integrity.
• Four weekly sessions of rESWT produce clinically meaningful improvements that persist four weeks post-treatment without additional rehabilitation interventions.
• This sham-controlled trial provides Level I evidence supporting rESWT as a cost-effective, non-invasive alternative for upper limb rehabilitation in chronic stroke.
• The dissociation between functional improvement and absent cortical MEPs suggests rESWT acts via peripheral neuromuscular modulation rather than central neuroplasticity.

## Introduction

Stroke is the second leading cause of death in the world and the third-leading cause of death and disability combined.^1^ Post-stroke spasticity is a highly prevalent cause of disability in both upper and lower limbs.^2^^,^^3^ It is charactherized by a velocity-dependent increase in the tone of muscles and deep tendon reflexes. The pathopsysiology of post-stroke spasticity is complex involving the imbalance between excitatory and inhibitory mechanisms causing the hyperexcitability of the stretch reflex and increased muscle tone.

Extracorporeal shock wave therapy reduces spasticity [4-15] and restores lost motor functions of the upper limbs in stroke.^6^^,^^7^^,^^10^^,^^11^^,^^13^^,^^16^, ^17^, ^18^, ^19^ Radial Extracorporeal Shock Waves (rESWT) can be defined as single acoustic pulses characterized by high peak pressure (which can exceed 10 MPa), fast pressure rise (1 ms), short duration (approximately 20 ms), high energy density up to 0.45 mJ/mm^2^) and fast propagation in a three-dimensional manner.^20^ rESWT effects are possibly related to the destruction of end plates in the neuromuscular junction,^20^ reduction in the compound muscle action potential amplitude,^21^ and modulation of the release of nitric oxide at the neuromuscular junction.^22^ Even though the mechanisms of action of rESWT in spasticity are still not fully understood, its effects may be related to both a direct action at the peripheral nervous system and indirectly at the central nervous system.

Therefore, our objective was to demonstrate the effect of rESWT on functional recovery of the upper extremity after ischemic stroke in the middle cerebral artery territory. We hypothesized that rESWT, compared to sham, applied to spastic muscles may improve functional recovery after stroke. In addition, we tested the hypothesis that rESWT applied over spastic muscles may result in changes in cortical excitability measured with TMS.

## Materials and methods

This study is a randomized, double-blind, sham-controlled trial that evaluated the effect of rESWT on upper limb functional disability in chronic stroke patients. The research was conducted at the *Instituto de Medicina Fisica e Reabilitacao, Hospital das Clinicas HCFMUSP, Faculdade de Medicina, Universidade de São Paulo*, São Paulo, SP, Brazil, from August 2013 to May 2018.

The study was approved by the local Ethical Committee, *Comite de Analise de Projetos de Pesquisa (*CAPPesq), of the *Universidade de São Paulo*, registered with number 19109813.1.0000.0068, and all patients signed an informed consent form before any study procedure. The study is reported according to the guidelines for Good Clinical Practice and the Consolidated Standards of Reporting Trials (CONSORT).

A sham rESWT handpiece (that looked, felt, and sounded identical to a real rESWT handpiece but did not emit rESWT) was provided free of charge by Electro Medical Systems S.A. (Nyon, Switzerland). This trial is registered at the US National Library of Medicine #NCT02924168. It is important to note that the control group did not experience any tactile sensation and, for that reason, such sham-controlled RCTs on ESWT with blinded patients can only be conducted on patients who have never experienced ESWT before, which was the case in our study. Also, patients participating in this study never met and did not know who else was participating in this study.

### Subjects

The sample size was estimated based on a study by Manganotti and Amelio,^4^ who found an effect size of 0.58 for rESWT. Considering alpha of 0.05 and power of 0.80, the sample size of eight participants per study group was estimated. We added 20 % for possible losses, therefore, ten patients per intervention group were the final estimation.

The inclusion criteria of this study were: 18-years of age or above; clinical and radiological diagnosis with Magnetic Resonance Imaging (MRI) or Computed Tomography (CT) scan indicating stroke in the territory of the middle cerebral artery; 6 to 24-months from stroke ictus; clinical stability and written informed consent to participate in the study. The rationale behind these periods was to exclude acute and subacute patient populations as well as patients with lower recovery rates after two years post-stroke. Individuals were not included if they presented a score of less than 24 on the Mini-Mental test, previously documented strokes, pregnancy, previous treatment with botulinum toxin, phenol alcohol or surgery for six months prior to inclusion, bone diseases, pre-existing joint damage or limb deformities that would limit the implementation of the proposed therapy; psychoactive disorders that would prevent adherence or joint pain that would occur within the movement range of therapies; and contraindications of rESWT.

Patients were discontinued if any of the following occurred: injuries and/or pain at the joint, muscle, or tendon that hindered or prevented the realization of movements; progressive worsening of spasticity (reaching level 4 on the Ashworth Scale); consent withdrawal; new episodes of stroke and uncontrolled hypertension, diabetes or seizures; or concomitant treatment with botulinum toxin or phenol alcohol.

### Blinding and randomization

All patients were randomly assigned to receive either rESWT (treated group) or sham rESWT (control group). Randomization was performed using a computerized random number generator. The randomization numbers were placed in sealed opaque envelopes, and allocation to groups was concealed from the patients until the end of the study and from the physicians applying the therapy until treatment started. Evaluations were performed by an examiner who was unaware of the treatment allocations. Patients in the control group identically received sham treatment as the patients in the rESWT group, except that no real rESWT was applied, as no energy was transmitted.

### Assessments and intervention

Patients were scheduled to attend the hospital for six visits. During the screening visit, the baseline clinical characteristics were assessed, the patients’ history was recorded, and the patients signed the Informed Consent form. During the second visit, the patients undertook baseline functionality assessments, TMS evaluations, and received the first application of rESWT or sham rESWT. Visits 3 and 4 were only used for applying rESWT or sham rESWT. The participants received the last (4^th^) intervention application at the fifth visit, followed by the study evaluations. Four weeks after the last intervention session, the patients had their final visit and were evaluated for functionality and TMS.

### Baseline clinical characteristics

On the first day of the study, a qualified physician recorded the patient’s history and performed a physical examination, in addition to reviewing inclusion and exclusion criteria. The patient’s history included age, gender, body height, body weight, ethnicity, schooling, medications in use, presence of comorbidities, and stroke characteristics (mechanism, number of events, dates of events, location).

We also selected instruments with the potential to evaluate the global characteristics of the stroke sequelae and the impact on the quality of life of these patients. In addition, tools were used to evaluate factors that could potentially influence the recovery of patients, such as cognitive, pain, and mood changes.

We used the Mini Mental State Examination (MMSE) for cognition screening. Mood changes were measured by the Hamilton Depression Scale (HDS). We used the National Institutes of Health Stroke Scale (NIHSS) to determine stroke characteristics. Finally, concerning the impact on quality of life and general functionality, we used the Stroke Impact Scale (SIS) and the Brazilian version of the Functional Independence Measure (FIM).

### Intervention

Patients were treated with four sessions of rESWT delivered with the Swiss Dolorclast device with the 36 mm applicator attached to the Power+ handpiece (EMS Electro Medical Systems S.A., Nyon, Switzerland). Each patient was treated one time per week for four weeks. Every patient received 5000 continuous rESWT per treatment session, diffusely in elbow and wrist flexors in the forearm and hand interosseus at 3.5‒4.0 bar air pressure with an Energy Density (ED) of approximately 0.07 mJ/mm^2^. The rESWT was applied at a frequency of 15 Hz. The air pressure (and, thus, ED) applied to each patient was adjusted according to the maximum intensity the patient could tolerate.

### Primary outcome measure

The primary outcome was the individual mean value in the Fugl-Meyer Assessment for Upper Extremity (FMA-UE) from baseline to four weeks after the last application of rESWT or sham rESWT. The FMA-UE is specifically designed to evaluate functional recovery in hemiplegic patients, and it is divided into five domains: motor function, sensitivity, balance, range of motion, and pain. The FMA-UE was assessed in its global score and partitioned in assessing the function of the proximal and distal muscle groups. Each item is scored as 0 (unable to perform), 1 (partial ability to perform), or 2 (near normal ability to perform). The total upper limb motor function score ranges from 0 to 66.^23^

For this study, we selected the motor function domain of the upper limb part of the FMA-UE, which includes measurement of movement, coordination, and reflex activity of the shoulder, elbow, wrist, and hand, as it has been shown to have excellent intra-rater reliability and high inter-rater reliability.^23^ Evaluators were well-trained to standardize the scale's application and thus reduce evaluation subjectivity.

It was belatedly realized that the treatment does not equally affect the proximal and distal parts of the upper limbs. For this reason, we propose to decompose the total FMA-UE scores into proximal and distal components. The proximal portion of FMA-UE considered section A without the reflex and the regular reflex activity (30-points total), while the distal portion included sections B and C (24-points total). The partial proximal and distal analysis did not include reflex activities, and coordination and speed, as these tests are not specific for proximal or distal portions of the upper extremities.

### Secondary outcome measures

The following measures were used to explore the patient’s functional recovery further. The Modified Ashworth Scale (MAS) was used to assess spasticity. It is classified into five categories: “no increase in tone” to “rigid in flexion or extension”, according to muscle resistance against passive movement. Regarding the upper limb, the abduction of the shoulder, flexion and extension of the elbow, and extension of the wrist and fingers were evaluated.

The Medical Research Council Scale (MRC) is a rapid assessment that ranks muscle strength into six levels. This study evaluated active abduction of the shoulder, flexion and extension of the elbow, pronation and supination of the forearm, and flexion and extension of the wrist and fingers.

Grip and Pinch Strength (GPS), like the MRC, has the purpose of evaluating muscular strength, however, with greater sensitivity, since a continuous measurement is used with the kilogram-force scale, as measured by a specific dynamometer for palmar grip and another for pinch strength.

For the neurophysiological evaluation, corticospinal excitability measurements with TMS were performed (single pulse and paired). TMS was performed with a Magstim Rapid stimulator (The Magstim Company, Whitland, UK) using an eight-shaped 70 mm coil. The response to the stimulus applied to the motor cortex was registered using localization of the contralateral muscles. Hydrochloride silver electrodes were placed on the muscle belly (1^st^ interosseous) (active electrode) and over the muscle tendon or joint (reference electrode) to register the Motor-Evoked Potential (MEP). A ground electrode was positioned on the handle.

Motor Evoked Potentials (MEPs) were measured for Cortical Inhibition (ICI), Intracortical Facilitation (ICF), and duration of Cortical Silent Period (CSP). The motor threshold is the minimum intensity required to extract an MEP of 50 or 100 microvolts in amplitude as determined by peak-to-peak. All TMS assessments were conducted in the morning.

### Evaluation routine

All evaluations of the primary and secondary outcomes were assessed immediately before the first application of rESWT or sham rESWT, immediately after the first application, as well as four weeks after the last application of rESWT or sham rESWT (i.e., eight weeks post-baseline).

### Statistical analysis

The main interest of this research is to assess the durable effect of radial shock waves after four weeks of the last intervention.

This type of pre-post design is typically evaluated using a *t*-test or, equivalently, a one-way ANOVA to assess the differences. Among these fundamental tests, the Student’s *t*-test assumes univariate normality of the Dependent Variable (DV) in each condition and homoscedasticity between the two conditions. In contrast, Welch-Satterthwaite’s *t*-test does not require homoscedasticity. Both tests have the limitation of lacking control over the baseline, which is determined by the starting point of the patients.

It can be circumvented by the Analysis of Covariance (ANCOVA), which can control the baseline level as a covariate. For ANCOVA, the outcome after four weeks of the last intervention is the Dependent Variable (DV), the treatment (rESWT or sham rESWT) is the Independent Variable (IV), and the measurement taken on the first day of the study is assumed as the baseline and included as a Covariate (CV). Both ANOVA and ANCOVA are implemented as linear regression models. The main assumptions were tested: (a) Regression homoscedasticity using Breusch-Pagan tests, (b) Linear relationship between CV and DV at each level of IV using the Harvey-Collier test, (c) Bivariate normality using Henze-Zirkler test, and (d) Univariate normality of CV and DV at each level of IV by Shapiro-Wilk. Details are described in the “Results, general assumptions” section. Additional assumptions for ANCOVA were also verified, namely (a) Dissociation between IV and CV and (b) The parallelism of linear regressions between CV and DV for each level of IV (slope homogeneity). When these regressions are assumed as parallel lines, the distance between them estimates the effect of IV (the null hypothesis is that distance equals zero between intercepts or centroids). Details of this method are in the “Results, ANCOVA” section.

A drawback of ANCOVA is that the intercept difference cannot be interpreted when slope homogeneity is rejected and, therefore, the regression lines are not parallel between the patient groups. In order to double-check the existence of the treatment effect, it was also approached using Hotelling’s *T*^2^ test, equivalent to a Multivariate Analysis of Variance (MANOVA) when only two groups are compared. This test does not require slope homogeneity but requires multivariate homoscedasticity, which is tested by Box’s M. The last improvement of this test is Johansen’s *T*^2^, which is robust to the lack of slope homogeneity and multivariate homoscedasticity. Both methods are implemented in the *R* package SHT. Details of these methods are in the “Results, Hotelling’s and Johansen’s *T*^2^” section. All analyses consider an effect statistically significant if its associated p-value is smaller than 0.05.

## Results

**Fig. 1:**
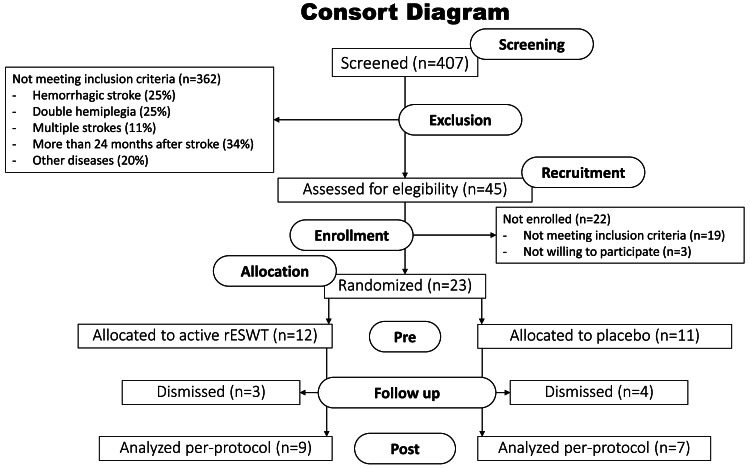
Consort flow: inclusions, allocation, treatment, and analysis (rESWT, Radial Extracorporeal Shockwave Therapy).

From August 2013 to January 2018, 407 patients from the Hemiplegia Outpatient Clinic of the Physical Medicine and Rehabilitation Institute of the Hospital das Clínicas (IMREA) were screened for study eligibility. From this group, 45 patients were recruited, however 22 were not included (19 for not meeting the inclusion criteria and 3 for declining participation). Finally, 23 patients with the diagnosis of ischemic stroke in the middle cerebral artery territory were included and randomized 1:1 in one of both treatment groups: Extracorporeal Shock Wave Therapy group (rESWT) or placebo (sham rESWT). Three patients of the rESWT group and four patients of the sham rESWT group were excluded due to the use of Botulinum toxin, declined participation soon after they were randomized, or did not return four weeks after the end of treatment to follow-up. Therefore, 16 patients were included in the per-protocol analysis. The patients received no other intervention except for rESWT during the study period. The flow of inclusion, allocation, and analysis is shown in Fig. 1. A significant finding at baseline was that all recruited patients (23/23) presented with absent Motor Evoked Potentials (MEPs) in the affected hemisphere, indicating severe corticospinal tract damage.

**Fig. 2:**
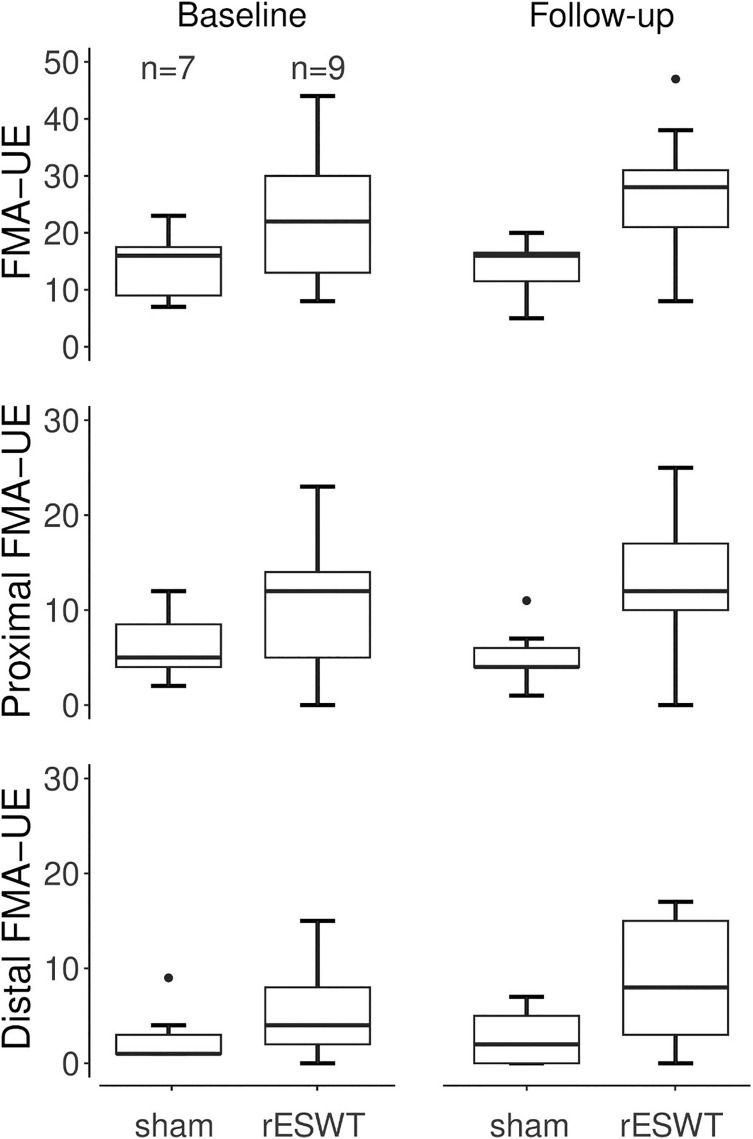
Fugl-Meyer for upper limb assessment (FMA-UE) of both groups at baseline and four weeks after the last intervention session (eight weeks after the beginning of the treatment).

**Fig. 3:**
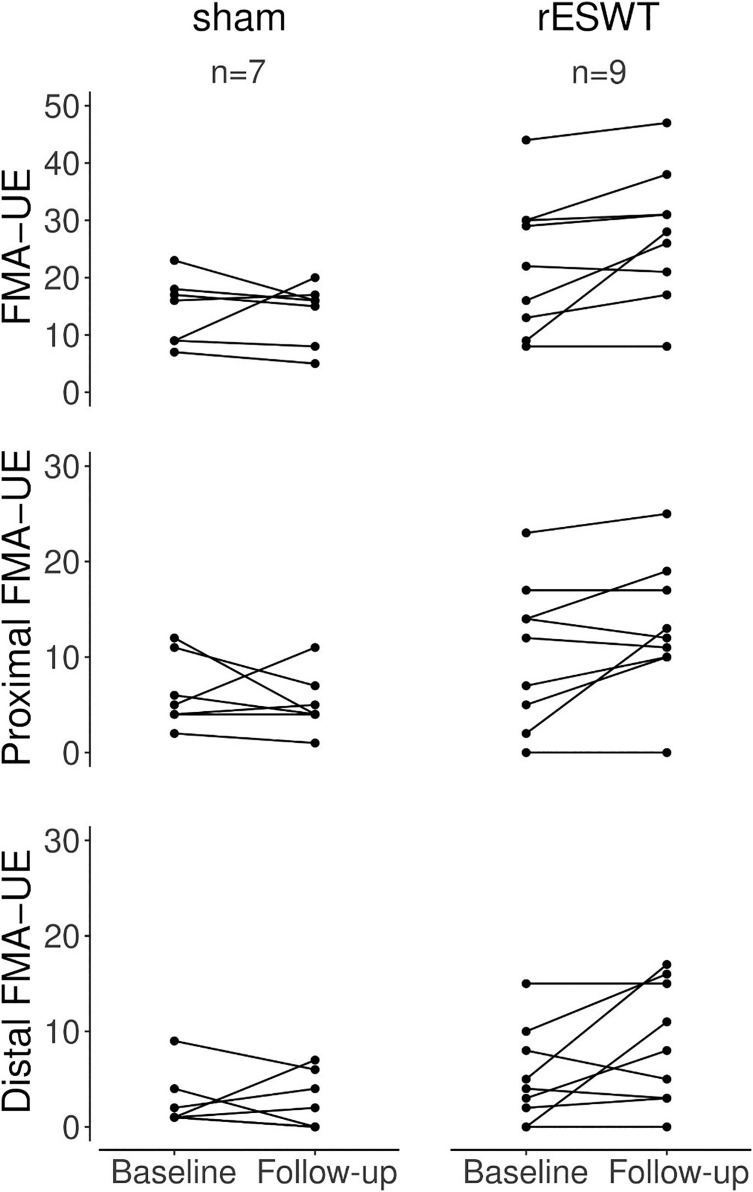
Individual Fugl-Meyer for upper limb assessment (FMA-UE) measures at baseline and four weeks after the last intervention session (eight weeks after the beginning of the treatment).

**Fig. 4:**
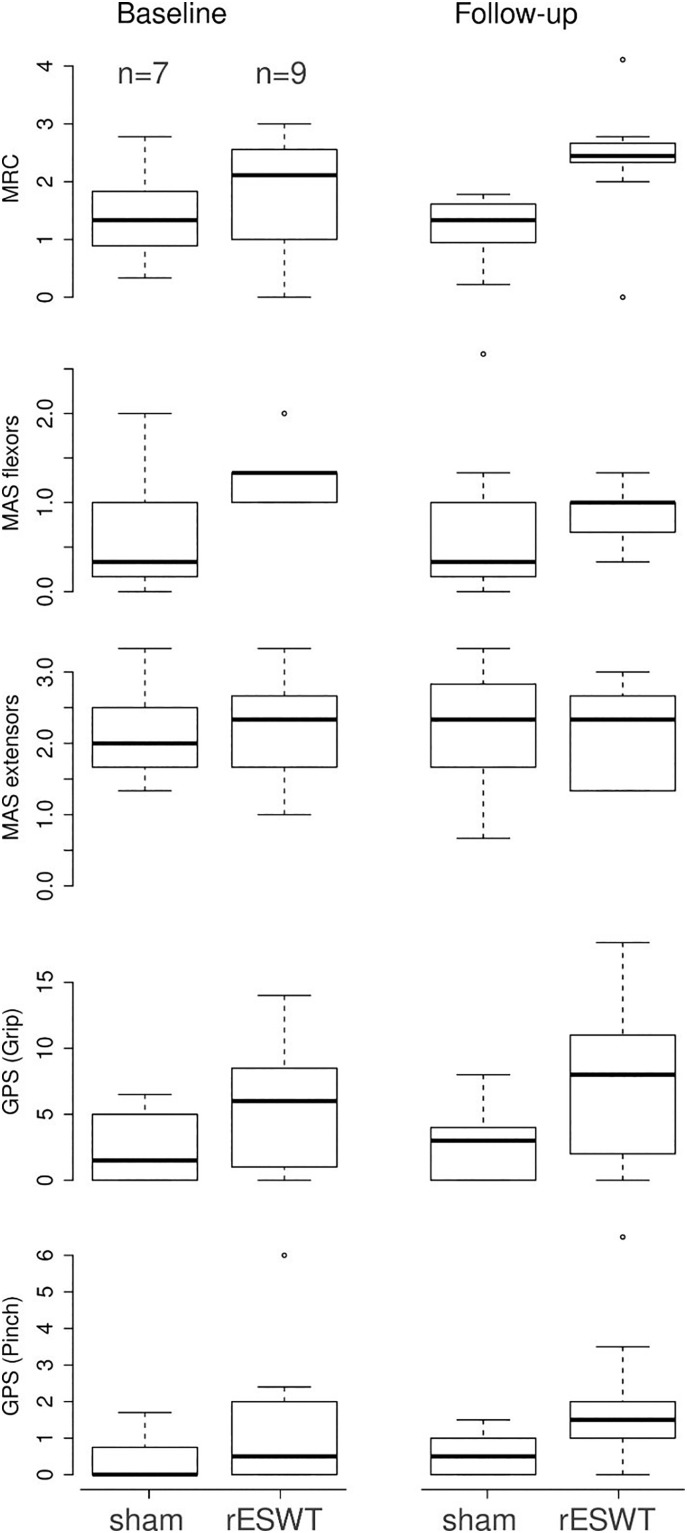
Secondary outcomes of both groups at baseline and four weeks after the last intervention session (eight weeks after the beginning of the treatment). MRC, Medical Research Council Scale; MAS, Modified Ashworth Scale (flexor and extensor muscles); GPS, Grip and Pinch Strength.

The demographic and values of the primary outcome (Fugl-Meyer Assessment for Upper Extremity) of both intervention groups of patients are shown in Table 1. Baseline characteristics of these patients are shown in Table 2. Table 3 shows that rESWT and sham rESWT groups were comparable at the baseline conditions (patients with missed follow-up were excluded from the analysis). Fig. 2, Fig. 3 show the sample values of the primary outcome of all patients included in the analysis. Fig. 4 shows the sample distribution of secondary outcomes.

**Table 1:** Characteristics of patients and primary outcome measure (raw data). Patients with missed follow-up were excluded from the subsequent analysis.

**ID**	**Group**	**Sex**	**Age**	**Stroke**	**FMA-UE (total score)**		**Proximal FMA-EU**		**Distal FMA-UE**	
					**Baseline**	**After treatment**	**Baseline**	**After treatment**	**Baseline**	**After treatment**
1	rESWT	Male	43.5	18.8	22	21	14	12	4	3
3	rESWT	Male	47.6	22.7	44	47	23	25	15	15
4	rESWT	Male	35.5	17.6	10	Missed	0	Missed	2	Missed
7	rESWT	Female	72.3	4.8	8	8	0	0	0	0
9	rESWT	Male	40.7	6.6	16	26	5	10	3	8
10	rESWT	Female	75.1	7.9	9	28	2	13	0	11
13	rESWT	Female	53.2	15.3	29	31	12	11	10	16
14	rESWT	Male	26.7	21.6	24	Missed	10	Missed	6	Missed
15	rESWT	Male	31.6	19.6	30	38	17	17	5	17
20	rESWT	Female	43.8	16.4	30	31	14	19	8	5
21	rESWT	Male	37.4	11.0	26	Missed	17	Missed	2	Missed
23	rESWT	Male	46.1	14.4	13	17	7	10	2	3
		**Mean**	46.1	14.7	21.8	27.4	10.1	4.8	13.0	8.7
		**std. dev.**	14.8	5.9	10.9	11.5	7.4	4.4	7.0	6.3
2	sham	Female	67.8	6.1	9	8	4	4	1	0
56	sham	Female	77.2	24.7	9	Missed	5	Missed	0	missed
	sham	Male	65.6	23.9	4	Missed	2	Missed	0	Missed
8	sham	Male	40.7	9.6	16	17	11	7	1	2
11	sham	Male	64.8	15.2	9	20	4	5	1	7
12	sham	Female	74.3	21.6	12	Missed	8	Missed	0	Missed
16	sham	Female	69.3	16.9	23	16	6	4	9	6
17	sham	Female	66.3	19.2	18	16	12	4	2	4
18	sham	Female	38.5	16.8	24	Missed	12	Missed	8	Missed
19	sham	Female	57.3	18.6	17	15	5	11	4	0
22	sham	Female	54.7	7.0	7	5	2	1	1	0
		**Mean**	61.5	16.3	13.5	13.9	6.5	2.5	5.1	2.7
		**std. dev.**	12.6	6.4	6.6	5.3	3.8	3.2	3.1	3.0

FMA-UE, Fugl-Meyer Assessment for Upper Extremety; Age, Years; Stroke, Time between stroke and treatment (months); rESWT, Treated group; sham, Placebo rESWT group.

**Table 2:** Baseline clinical characteristics of patients.

**ID**	**Treatment**	**MMSE**	**HDS**	**SIS**	**NIHSS**	**FIM**
1	rESWT	30	3	73	4	73
3	rESWT	27	2	70	3	78
4	rESWT	30	6	68	5	73
7	rESWT	20	13	22	15	37
9	rESWT	10	8	52	9	76
10	rESWT	5	12	41	12	65
13	rESWT	23	9	57	11	74
14	rESWT	24	5	62	6	78
15	rESWT	28	1	83	6	78
20	rESWT	24	4	41	5	79
21	rESWT	27	1	68	1	77
23	rESWT	20	8	55	8	77
	**Mean**	22.3	6.0	57.7	7.1	72.1
	**std. dev.**	7.8	4.1	16.9	4.1	11.7
2	sham	12	1	62	7	63
5	sham	14	23	22	11	51
6	sham	26	23	39	13	19
8	sham	26	12	64	4	57
11	sham	29	0	60	6	76
12	sham	20	6	42	7	70
16	sham	30	9	53	6	70
17	sham	20	14	43	5	71
18	sham	28	6	59	6	73
19	sham	21	6	68	5	78
22	sham	27	19	50	9	39
	**mean**	23.0	10.8	51.1	7.2	60.6
	**std. dev.**	6.1	8.1	13.6	2.8	18.1

MMSE, Mini-Mental State Examination; HDS, Hamilton Depression Scale; SIS, Stroke Impact Scale; FIM, Brazilian version of the Functional Independence Measure; NIHSS, United States National Institutes of Health Stroke Scale; rESWT, Treated group; sham, Placebo rESWT group.

**Table 3:** Baseline clinical characteristics per treatment of patients included in this study (patients without follow-up eight weeks after the first treatment session were excluded). Statistical comparison between groups by Welch two-sample *t-*test: rESWT (n = 9, male = 5, female = 4) and sham rESWT (n = 7, male = 2, female = 5).

**Characteristic**	**Treatment**	**Mean**	**SD**	**Med**	**Min**	**Max**	***t***	**Df**	**p**
Age	rESWT	50.44	14.42	46.09	31.65	75.15	1.575	13.91	0.138
	sham	60.13	10.16	64.81	40.68	69.32			
Time stroke	rESWT	14.06	6.27	15.33	4.83	22.70	–0.282	13.70	0.782
	sham	13.22	5.55	15.23	6.07	19.23			
MMSE	rESWT	20.78	8.35	23.00	5.00	30.00	0.760	14.00	0.460
	sham	23.57	6.35	26.00	12.00	30.00			
HDS	rESWT	6.67	4.36	8.00	1.00	13.00	0.684	9.59	0.510
	sham	8.71	6.92	9.00	0.00	19.00			
SIS	rESWT	54.75	18.72	55.43	21.95	82.84	0.359	11.91	0.726
	sham	57.28	8.80	60.19	43.12	67.93			
NIHSS	rESWT	8.11	4.01	8.00	3.00	15.00	–1.433	11.10	0.179
	sham	6.00	1.63	6.00	4.00	9.00			
FIM	rESWT	70.78	13.36	76.00	37.00	79.00	–0.874	12.97	0.398
	sham	64.86	13.51	70.00	39.00	78.00			
FMA-UE	rESWT	22.33	11.95	22.00	8.00	44.00	–1.794	12.20	0.098
	sham	14.14	5.90	16.00	7.00	23.00			
Prox.FMA-UE	rESWT	10.44	7.50	12.00	0.00	23.00	1.445	12.31	0.174
	sham	6.29	3.77	5.00	2.00	12.00			
Dist.FMA-UE	rESWT	5.22	4.97	4.00	0.00	15.00	1.251	13.32	0.232
	sham	2.71	2.98	1.00	1.00	9.00			
MRC	rESWT	1.80	1.07	2.11	0.00	3.00	0.823	14.00	0.424
	sham	1.41	0.82	1.33	0.33	2.78			
MAS flex.	rESWT	1.26	0.32	1.33	1.00	2.00	1.964	7.77	0.086
	sham	0.67	0.75	0.33	0.00	2.00			
MAS ext.	rESWT	2.26	0.83	2.33	1.00	3.33	0.306	13.90	0.764
	sham	2.14	0.69	2.00	1.33	3.33			
Grip	rESWT	5.61	5.25	6.00	0.00	14.00	1.473	12.86	0.165
	sham	2.57	2.89	1.50	0.00	6.50			
Pinch	rESWT	1.32	1.98	0.50	0.00	6.00	1.226	10.23	0.248
	sham	0.46	0.67	0.00	0.00	1.70			

Age, Years; Time stroke, Time between stroke and treatment in months; MMSE, Mini-Mental State Examination; HDS, Hamilton Depression Scale; SIS, Stroke Impact Scale; NIHSS, National Institutes of Health Stroke Scale; FIM, Functional Independence Measure; FMA-UE, Fugl-Meyer Assessment for Upper Extremity (global, proximal and distal); MRC, Medical Research Council Scale; MAS, Modified Ashworth Scale for flexor and extensor muscles; Grip, Grip strength; Pinch, Pinch strength; rESWT, Treated group; Sham, Placebo rESWT group; SD, Standard deviation, Med, Median; Min: minimum value; Max, Maximum value; *t*, test statistics; Df, Degrees of freedom; p, p-value (there is no significant differences between rESWT and sham rESWT groups).

### Primary outcome (FMA-UE)

The raw data of the primary outcome and the mean and standard deviation of each group are presented in Table 1. It can be observed that the mean and standard deviation of global, proximal, and distal values of FMA-UE are numerically higher in the rESWT group compared to the sham rESWT group at both baseline and four weeks after the last treatment session. The two groups start with numerically different values and a small sample size, so special statistical procedures are required to account for the baseline when assessing the treatment effect.

### General assumptions

Several assumptions tests required by inferential statistics were conducted. Box’s M for homogeneity of covariance matrices is a multivariate test of homoscedasticity; it compares rESWT and sham rESWT covariance matrices obtained from the correlation between FMA-UE at the beginning of the study (base) and after four weeks of the last intervention (after). Breusch-Pagan has the null hypothesis that the points are homogeneously distributed along the regression line (regression homoscedasticity) in each condition (rESWT or sham rESWT). Harvey-Collier has the null hypothesis of a linear relationship between the FMA-UE base and after in each condition. Finally, multivariate normality is assessed using the Henze-Zirkler test, and univariate normality is assessed using Shapiro-Wilk. In all cases, the respective null hypotheses were not rejected (p > 0.05), except for the normality test for the sham rESWT group of Distal FMA-UE four weeks after the last treatment session (*p* = 0.002), which has secondary importance. Therefore, all linear regression models assumed homoscedasticity, linearity, and normality.

### t-test

Two versions of the *t-*test, namely the traditional Student’s *t-*test and the Welch-Satterthwaite’s correction, were performed. These tests are equivalent to a one-way ANOVA applied to two independent conditions. While the Student’s *t-*test assumes homoscedasticity of the Dependent Variable (DV), which was not rejected (Breusch-Pagan test), Welch-Satterthwaite’s *t-*test can account for small numeric heteroscedasticity and is applicable. The null hypothesis tested is the absence of a difference between FMA-UE four weeks after the last treatment session and FMA-UE before the beginning of the treatment, both globally and separately for the proximal and distal muscle groups, and the results indicated no statistically significant differences (p > 0.05).

However, These traditional approaches do not consider the individual departing points (i.e., the individual values of FMA-UE of each patient at the beginning of the treatment), for they are masked by the difference after-base computation. For the present study, the weakness of these *t-*tests may preclude the detection of treatment effect.

### ANCOVA

ANCOVA can include the baseline FMA-UE as a covariate to better control the change to FMA-UE four weeks after the last treatment session. In addition to *t-*tests, its assumptions are the null hypothesis of dissociation between the treatment group and FMA-UE base (not rejected for global, proximal, and distal muscle groups) and the parallelism between linear regressions for both treatments (also not rejected). The null hypothesis of parallel regression lines is not rejected when it is possible to draw two parallel lines representing the populational linear regressions, each located inside its respective confidence band, which was true for the three FMA-UE measures.

Assuming that both regressions are parallel lines, the null hypothesis of the null difference between intercepts is rejected for the global FMA-UE (*p* = 0.038) and the proximal component of FMS-UE (*p* = 0.030) shown in Table 5 leading to the conclusion of the existence of treatment effect.

### Hotelling’s and Johansen’s T^2^

For these tests, the linear regressions are computed with different slopes (there is no assumption of parallelism nor estimate of common slope). The translation of both centroids follows the exact mechanism of ANCOVA. The null hypothesis posits an Euclidean statistical distance of zero between the translated centroids.

Hotelling’s *T*^2^ test mainly assumes multivariate homoscedasticity (Box’s M) and bivariate normality (Henze-Zirkler). Johansen’s *T*^2^ only requires the latter. None of the assumptions were rejected.

Using both approaches, we reject the null hypothesis of the absence of distance between population centroids. The analytical method reveals this rejection through significant *p* values showing the treatment effect.

### Secondary outcomes

Table 4 contains raw data on all secondary outcomes (including patients with a lack of follow-up). For MAS of the flexor muscles, the null hypothesis of bivariate homoscedasticity is rejected, hindering Hotelling’s *T*^2^, and the univariate normality was rejected only in the rESWT group after the treatment, hindering *t-*tests and ANCOVA. In addition, the verification of dissociation between the group and baseline and parallelism of regressions were also rejected (Table 5). Therefore, the most reliable result for MAS of flexor muscles is Johansen’s *T*^2^, which does not show a difference between groups.

**Table 4:** Secondary outcome measures (raw data). Patients marked with a gray background were excluded from this study due to the absence of follow-up after four weeks of the last treatment session.

ID	Group	MRC (total score)		MAS (flexion)		MAS (extension)		Grip strength		Pinch strength	
		Baseline	Post-treatment	Baseline	Post-treatment	Baseline	Post-treatment	Baseline	Post-treatment	Baseline	Post-treatment
1	rESWT	2.44	2.33	1.33	1.00	3.33	2.67	8.00	8.00	0.00	2.00
3	rESWT	3.00	4.11	1.00	1.00	1.67	1.33	14.00	18.00	6.00	6.50
4	rESWT	2.11		0.33		2.33		5.00		2.50	
7	rESWT	0.00	0.00	2.00	0.50	2.67	3.00	0.00	0.00	0.00	0.00
9	rESWT	1.89	2.67	1.00	0.67	2.67	2.67	1.00	6.00	0.50	1.25
10	rESWT	0.44	2.33	1.00	1.00	1.00	1.33	0.00	2.00	0.00	1.50
13	rESWT	2.11	2.78	1.33	0.33	3.33	2.00	1.50	2.00	0.00	1.00
14	rESWT	1.56		0.33		2.33		12.00		4.00	
15	rESWT	2.78	2.44	1.33	1.33	1.33	2.33	11.50	13.00	2.00	1.00
20	rESWT	2.56	2.67	1.00	1.00	2.33	1.33	6.00	11.00	1.00	1.70
21	rESWT	2.11		1.00		3.33		20.00		3.50	
23	rESWT	1.00	2.00	1.33	0.67	2.00	2.67	8.50	8.00	2.40	3.50
2	sham	0.67	0.22	0.67	0.67	2.33	2.67	0.00	0.00	0.00	0.00
5	sham	0.89									
6	sham	0.67									
8	sham	1.89	1.78	1.33	1.33	3.33	3.00	0.00	2.00	0.00	0.00
11	sham	1.78	1.33	0.00	0.00	1.33	0.67	6.00	8.00	1.70	1.50
12	sham	1.44		1.50		3.33		1.00		1.00	
16	sham	2.78	1.67	0.33	0.33	2.00	2.33	4.00	4.00	0.50	1.00
17	sham	1.11	1.56	2.00	2.67	2.67	3.33	1.50	4.00	0.00	1.00
18	sham	1.78		1.00		3.67		4.00		0.00	
19	sham	1.33	1.33	0.33	0.00	1.67	1.67	6.50	missing	1.00	missing
22	sham	0.33	0.56	0.00	0.33	1.67	1.67	0.00	0.00	0.00	0.00

MRC, Medical Research Council Scale; MAS, Modified Ashworth Scale (flexor and extensor muscles); Grip and Pinch Strength; Post-treatment, follow-up, four weeks after last intervention measure (eight weeks after treatment beginning); rESWT, Treated group; sham, Placebo rESWT group.

**Table 5:** Treatment effect, comparison between sham rESWT and rESWT groups (primary and secondary outcomes of patients included in the study).

	Sham difference	rESWT Difference	Student *t*	Welch *t*
**FMA-UE**	–0.286	5.111	*t*(14) = 1.784	*t*(13.8) = 1.817
(after treatment ‒ baseline)	(13.857 − 14.143)	(27.444 − 22.333)	*p* = 0.096	*p* = 0.091
**Prox. FMA-UE**	–1.143	2.556	*t*(14) = 1.761	*t*(12.5) = 1.744
(after treatment ‒ baseline)	(5.143 − 6.286)	(13.000 − 10.444)	*p* = 0.100	*p* = 0.106
**Dist. FMA-UE**	0.000	3.444	*t*(14) = 1.481	*t*(13.5) = 1.569
(after treatment ‒ baseline)	(2.714 − 2.714)	(8.667 − 5.222)	*p* = 0.161	*p* = 0.140
**MRC**	–0.206	0.568	*t*(14) = 2.413	*t*(14.0) = 2.517
(after treatment ‒ baseline)	(1.206 − 1.413)	(2.370 − 1.802)	***p* = 0.030**	***p* = 0.025**
**MAS flexor**	0.095	–0.426	*t*(14) = –2.277	*t*(13.3) = –2.428
(after treatment ‒ baseline)	(0.762 − 0.667)	(0.833 − 1.259)	***p* = 0.039**	***p* = 0.030**
**MAS extensor**	0.048	–0.111	*t*(14) = –0.477	*t*(13.1) = –0.511
(after treatment ‒ baseline)	(2.190 − 2.143)	(2.148 − 2.259)	*p* = 0.640	*p* = 0.618
**Grip**	1.083	1.944	*t*(13) = 0.869	*t*(12.7) = 0.976
(after treatment ‒ baseline)	(3.000 − 2.571)	(7.556 − 5.611)	*p* = 0.400	*p* = 0.347
**Pinch**	0.217	0.728	*t*(13) = 1.321	*t*(12.5) = 1.495
(after treatment ‒ baseline)	(0.583 − 0.457)	(2.050 − 1.322)	*p* = 0.209	*p* = 0.160
	**ANCOVA assumptions**		**EMM**	**ANCOVA**
	**Dissociation**	**Parallelism**	**Difference**	**Effect**
**FMA-UE**	*t*(14) = 1.655	*t*(12) = 0.796	7.319	*t*(13) = 2.310
	*p* = 0.120	*p* = 0.442		***p* = 0.038**
**Prox. FMA-UE**	*t*(14) = 1.334	*t*(12) = 1.374	4.987	*t*(13) = 2.427
	*p* = 0.203	*p* = 0.195		***p* = 0.030**
**Dist. FMA-UE**	*t*(14) = 1.175	*t*(12) = 0.511	4.313	*t*(13) = 1.798
	*p* = 0.259	*p* = 0.618		*p* = 0.095
**MRC**	*t*(14) = 0.795	*t*(12) = 0.588	0.888	*t*(13) = 2.910
	*p* = 0.440	*p* = 0.567		***p* = 0.012**
**MAS flexor**	*t*(14) = 2.154	*t*(12) = –4.477	–0.465	*t*(13) = –1.708
	***p* = 0.049**	***p* = 0.001**		*p* = 0.111
**MAS extensor**	*t*(14) = 0.299	*t*(12) = –1.917	–0.118	*t*(13) = –0.374
	*p* = 0.769	*p* = 0.079		*p* = 0.714
**Grip**	*t*(14) = 1.372	*t*(11) = –0.189	0.717	*t*(12) = 0.639
	*p* = 0.192	*p* = 0.853		*p* = 0.535
**Pinch**	*t*(14) = 1.102	*t*(11) = 0.226	0.639	*t*(12) = 1.586
	*p* = 0.289	*p* = 0.825		*p* = 0.139
	**EMM**	**Hotelling’s***T*^2^	**Johansen’s***T*^2^	
	**Difference**	**Effect**	**Effect**	
**FMA-UE**	8.486	*T*^2^(2,15) = 9.235	*T*^2^ (2,15) = 9.770	
	***p* = 0.037**		***p* = 0.033**	
**Prox. FMA-UE**	5.996	*T*^2^(2,15) = 10.341	*T*^2^ (2,15) = 10.537	
	***p* = 0.027**		***p* = 0.028**	
**Dist. FMA-UE**	4.636	*T*^2^(2,15) = 4.418	*T*^2^(2,15) = 5.089	
	*p* = 0.168		*p* = 0.134	
**MRC**	0.909	*T*^2^(2,15) = 9.996	*T*^2^(2,15) = 11.465	
	***p* = 0.030**		***p* = 0.021**	
**MAS flexor**	–0.237	*T*^2^(2,15) = 1.084	*T*^2^(2,15) = 1.218	
	*p* = 0.616		*p* = 0.584	
**MAS extensor**	–0.139	*T*^2^(2,15) = 0.209	*T*^2^(2,15) = 0.219	
	*p* = 0.908		*p* = 0.903	
**Grip**	0.580	*T*^2^(2,14) = 0.090	*T*^2^(2,14) = 0.118	
	*p* = 0.960		*p* = 0.946	
**Pinch**	0.699	*T*^2^(2,14) = 2.829	*T*^2^(2,14) = 3.651	
	*p* = 0.307		*p* = 0.228	

Primary outcome: FMA-UE, Fugl-Meyer assessment for Upper Extremities. Secondary outcomes: MRC, Medical Research Council Scale; MAS, Modified Ashworth Scale for flexor and extensor muscles; Grip and Pinch Strength. Statistical tests, For each outcome, differences were calculated as follow-up minus baseline (follow-up assessed four weeks after the last session). Treatment effects were examined using complementary statistical approaches. In the top panel, independent-sample *t*-tests (Student’s *t*-test and Welch-Satterthwaite *t*-test) compared change scores between the rESWT and sham groups, with the Welch test accounting for potential heteroscedasticity. In the middle panel, ANCOVA assessed treatment effects on follow-up outcomes adjusted for baseline values, under the assumptions of independence between baseline and treatment group (dissociation) and homogeneity of regression slopes (parallelism), with effects estimated as differences in adjusted Estimated Marginal Means (EMMs) and their 95 % Confidence Intervals. In the bottom panel, multivariate *T^2^*-based methods (Hotelling’s *T²* and Johansen’s *T²*) evaluated treatment effects without assuming parallel regression slopes, using 95 % confidence ellipses centered on EMMs derived from non-parallel regression models.

The univariate normality was rejected for Pinch strength in three out of four subgroups. In addition, the linearity test was not computable for the sham rESWT group with measures of Grip and Pinch strengths. It hinders all proposed tests for univariate normality, an assumption for the *t-*tests, while linearity is important for ANCOVA and both *T*^2^ to find the estimated marginal means. It is observed in Table 4 that Pinch values have several repeated zeros on the sham group. Their results may be seen as inconclusive. Grip strength could not converge on the linearity test with the sham group. The *t-*test, although formally acceptable, does not use the baseline, as discussed above. Their results must also be seen with caution.

Besides global, proximal, and distal FMA-UL, Table 5 also summarizes the effects of treatment on these secondary outcomes. In general, there are also no significant changes in secondary outcomes. The exception was the treatment effect on MRC according to *t-*tests, ANCOVA, Hotelling *T*^2^, and Johansen^2^.

## Discussion

The major finding of this study was that four weekly sessions of rESWT clinically improved proximal functional capacity measured by FMA-UE in patients with chronic ischemic stroke in the territory of the middle cerebral artery without an MEP detectable by TMS in the lesioned hemisphere at baseline.

The treatment effect was examined using four different statistical methods. If we had only relied on *t-*tests and ANCOVA, the former would not have revealed a significant difference between the treatment conditions (rESWT) and placebo (sham rESWT). At the same time, the latter yielded *p* = 0.038 (Table 5), creating uncertainty about the existence of the effect and pitting one result against the other. Therefore, it may be recommended to include tests based on different assumptions to break the tie or resolve the ambiguity. Thus, it is considered that the Student’s *t-*test and Welch-Satterthwaite *t-*test require univariate normality of the treatment results. ANCOVA adds independence between baseline and treatment conditions and parallelism of regressions between baseline and the final outcome in both conditions.

On the other hand, Hotelling’s *T^2^* procedure does not require parallelism and assumes bivariate normality and homoscedasticity between the baseline and the outcome in each condition. Johansen’s *T*^2^, a more recent enhancement, does not require bivariate homoscedasticity. Interestingly, both versions of *T*^2^ were consistent with the ANCOVA result, with very close p values (*p* = 0.037 and *p* = 0.033, respectively, Table 5). Despite the small sample size, obtaining similar results with different and more robust approaches reinforces the evidence of the rESWT treatment effect on the observed FMA-UE scores.

In agreement with our findings, recent trials have demonstrated significant improvement in functional upper limb recovery after rESWT measured by FMA scores.^6^^,^^7^^,^^10^^,^^11^^,^^13^^,^^16^, ^17^, ^18^, ^19^ However, despite statistically significant improvement of motor function assessed by the FMA-UE score of 1.26 weighted mean difference (95 % CI 0.19–2.24; *p* = 0.01), there is high heterogeneity in the included studies.^11^ Even for the management of spasticity,^11^^,^^13^, ^14^, ^15^ high heterogeneity among different studies calls for more well-designed and conducted studies to confirm the benefits of ESWT for the management of people with stroke for various treatment regimes, including ESWT energy, number of impulses and treated target muscles; different controls such as botulinum toxin,^19^ associated rehabilitation interventions, use of electrical stimulation, oral antispastic drugs and placebo.

To our knowledge, this is the first study using functional recovery as a primary outcome for stroke patients in the Americas. All previous data was derived from patients managed in China,^10^^,^^16^^,^^17^^,^^19^ Korea,^5^^,^^18^ Egypt,^6^ and Sri Lanka.^7^ We highlight the relevance of the generalized population for future meta-analyses and encourage researchers from different regions to assess the functional benefits of rESWT.

Some interesting facts must be noted. All patients in our study had undetectable MEP in the lesioned hemisphere at baseline TMS assessment. This unexpected finding may explain the selected motor proximal improvements rather than the distal functioning of the upper extremities. It is essential to highlight that patients with chronic stroke patients with no MEP are not expected to improve their functioning status with conventional interventions.^24^ Our inclusion criteria were not intended to screen this patient population before randomization but after patient inclusion. A recent study showed that MT measured in the lesioned hemisphere by TMS strongly predicts motor function recovery, as a positive correlation exists between MT and FMA scores.^25^ A large volume of lesions in the corticomotor pathways are likely related to the absence of MEP, which in theory is related to the worse prognosis and the lack of recovery of motor function. The sensitive analysis showed that the patients in the active group did not have an improvement in motor function in the distal segment (region evaluated with TMS). However, in the proximal segment of the upper limb (not evaluated with TMS), this result is consistent with the literature.^25^ We cannot exclude the possibility of distal upper limb functional recoveries in stroke patients with motor thresholds during the TMS studies. Our study assessed a novel patient population, addressing an important clinical need. Interestingly, this subgroup of patients with absent Motor Evoked Potentials (MEPs) are traditionally of poor prognosis. Therefore, despite technical challenges, TMS MT could be considered a screening test for motor recovery in stroke patients. Unfortunatly, no previous studies evidenced effects in this more impaired patients. In this way, our study extend understanding in this population.

Interestingly, our spasticity assessment seems to disagree with previous studies, most demonstrating a significant reduction in spasticity in the short and long term.^6^, ^7^, ^8^^,^^12^^,^^26^ Our findings suggest that other mechanisms, rather than spasticity, may be involved in the functional recovery of middle artery ischemic stroke. In fact, we hypothesize that this finding might be related to changes in the rheological properties of spasticity, which can be measured in the FMA-UE. In animal models, the local peripheral effects of rESWT were described as possible destruction of end plates in the neuromuscular junction[20] and reduction in the compound muscle action potential amplitude.^21^ Similar to the current lack of understanding of the molecular and cellular mechanisms of ESWT mediating pain relief in knee OA, mechanisms involved in spasticity management reported by previous authors are hypothesized but are still not known.^14^

The literature showed that neurotransmission at the Neuromuscular Junction (NMJ) remained after shock wave application even after inducing degeneration of acetylcholine receptors, suggesting a transient dysfunction of nerve conduction at NMJs, which can be observed in later neurophysiological studies.^4^^,^^20^ These findings may explain a mechanism by which ESWT treatment might be effective for impaired limb muscle coordination, as it apparently reduces spasticity without peripheral neurological impairment.

We have also captured significant improvement in MCR values. As a secondary outcome, this finding is exploratory and should be considered carefully. However, improvement in muscle strength can be a feasible explanation for increased motor function in stroke patients. Also, it is interesting to notice that results obtained after the treatment regime delivered in four weeks are maintained up to four weeks after the end of the rESWT sessions intervention, without any other associated interventions. The number of cases included in this study was sufficient to identify a significant clinical effect of rESWT assessed by the FMA-UE scores. In summary, our study showed that rESWT significantly enhances muscle strength (MRC scale) and functional recovery (FMA-UE) through likely peripheral mechanisms, independent of corticospinal tract integrity. The dissociation between functional improvement and absent cortical MEPs suggests rESWT acts via peripheral neuromuscular modulation rather than central neuroplasticity. Further studies are required to identify the molecular and cellular mechanisms of ESWT mediating spasticity reduction in stroke patients and whether control of spasticity can be achieved together with motor functional recovery.

We have included only patients with middle cerebral ischemic stroke in order to limit other neurological and clinical confounders. We have also carefully assessed the most relevant confounders (personal factors), including depression, cognitive abilities, motor scores, pain, comorbidities, and motor thresholds. We strongly suggest that future studies include these relevant parameters, especially neurophysiological biomarkers, to identify the best treatment protocols for the best responders. We have also used sham control with no other associated interventions to avoid the confounding effects of combined interventions.

Unfortunately, we did not control for environmental air temperature, which could increase spasticity. However, the intervention was conducted in a room without windows, and we believe that the cold wind did not influence the results. Only one study controlled the environmental conditions.^9^ Future studies should be performed in the same seasons of the year. However, we did control for pain and other confounders. Additionally, it is also the first study to assess baseline neurophysiological predictors for motor recovery. Other strengths of our study include blinding of the physician who administered the therapy, RCT adherence to the CONSORT Statement, assessment of multiple clinical variables that could have influenced the response to the intervention and inclusion of their interference in our statistical analysis, low risk of selection, measurement and reporting bias. This sham-controlled trial provides Level I evidence supporting rESWT as a cost-effective, non-invasive alternative for upper limb rehabilitation in chronic stroke.

Further RCTs are warranted to determine whether even better results can be achieved with different rESWT protocols, such as increasing treatment sessions (e.g., five treatment sessions), adjusting frequencies (5 Hz), or increasing EFD (higher than 0.07). Besides investigating optimal treatment parameters, future studies could explore the effect of rESWT as a complementary approach that could be combined with central nervous system-targeted therapies for synergistic effects.

The most significant limitation of this study is its small sample size, which limits the statistical power and the generalizability of our findings. While we employed robust statistical methods to control for baseline characteristics, the results must be interpreted as preliminary and should be confirmed in a larger, multi-center trial. From a methodological and ethical standpoint, testing new treatments often involves a small number of patients, presenting additional challenges for statistical analysis. As a result of employing robust statistical methods and observing the convergence of their results, the conclusion supporting the treatment effect is strengthened. In essence, although this study faced a situation that poses difficulties for traditional methods of a pre-post experimental design with a small sample size, there is suggestive evidence for the beneficial effect of rESWT treatment measured by the improvement in FMA-UE scores.

Another limitation of our study is that the final follow-up examination was performed eight weeks after the beginning of the treatment and four weeks after the last session of rESWT, which was relatively soon after the treatment. Additionally, we did not use the presence of MT detectable by TMS as an inclusion criterion. At last, while a sham device without tactile sensation is the accepted standard for ESWT trials, we cannot fully discount the possibility that the lack of sensation in the control group may have influenced patient expectations, despite our precautions with ESWT-naïve participants. For future studies, we strongly recommend further exploration of functional outcomes, including TMS under different interventions and different neurological baseline settings.

The study's findings have important implications for clinical practice, particularly in resource-limited settings where cost-effective interventions are essential. Treatment regimen is feasible, and sustained effects are clinically meaningful. By providing evidence for a treatment option for patients who typically show limited progress, this research addresses an important unmet need in stroke care.

## Conclusion

Our results demonstrate that rESWT is an effective intervention for improving proximal upper limb function and muscle strength in patients with chronic ischemic stroke. Crucially, this benefit was achieved in individuals with absent MEPs at baseline, a subgroup with traditionally poor prognosis. This suggests that the therapeutic mechanism of rESWT may act via peripheral mechanisms, potentially involving modulation of muscle tone and rheological properties at the neuromuscular junction, rather than central neuroplasticity. Future large-scale trials should focus on standardizing optimal treatment protocols and investigating the long-term maintenance of these functional gains.

## Supporting information

All raw data and statistical R scripts developed for these analyses are available in Harvard Dataverse, accessible through https://doi.org/10.7910/DVN/1KQ3GE.

## CRediT authorship contribution statement

**Marta Imamura:** Conceptualization, Methodology, Validation, Investigation, Writing – original draft, Writing – review & editing. **Andre Tadeu Sugawara:** Conceptualization, Methodology, Validation, Investigation, Writing – original draft, Writing – review & editing. **Artur Cesar Aquino Santos:** Project administration, Visualization, Validation, Investigation, Writing – original draft, Writing – review & editing. **Jose Oliveira Siqueira:** Formal analysis, Writing – original draft, Writing – review & editing. **Paulo Sergio Panse Silveira:** Formal analysis, Writing – original draft, Writing – review & editing. **Christoph Schmitz:** Conceptualization, Visualization, Writing – original draft, Writing – review & editing. **Linamara Rizzo Battistella:** Resources, Funding acquisition, Data curation, Visualization, Supervision, Writing – original draft, Writing – review & editing.

## Declaration of competing interest

C.S. served until Dec/2017 and serves since Jul/2024 as consultant for Electro Medical Systems (Nyon, Switzerland), the inventor, manufacturer and distributor of the Swiss Dolorclast rESWT device. However, Electro Medical Systems did not have any role in the design of this study, data collection and analysis, interpretation of the data, decision to publish and writing the manuscript. The other authors declare no conflict of interest.

## Data Availability

The datasets used/or analyzed during the current study are available from the corresponding author upon reasonable request.
